# Applying the neuroscience of creativity to creativity training

**DOI:** 10.3389/fnhum.2013.00656

**Published:** 2013-10-16

**Authors:** Balder Onarheim, Morten Friis-Olivarius

**Affiliations:** ^1^Department of Management Engineering, Technical University of DenmarkCopenhagen, Denmark; ^2^Decision Neuroscience Research Group, Copenhagen Business SchoolCopenhagen, Denmark; ^3^Danish Research Center for Magnetic Resonance, Centre for Functional and Diagnostic Imaging and Research, Copenhagen University Hospital HvidovreHvidovre, Denmark

**Keywords:** creativity, neuroscience, psychology, neuroscience of creativity, neurocreativity, teaching, application, training

## Abstract

This article investigates how neuroscience in general, and neuroscience of creativity in particular, can be used in teaching “applied creativity” and the usefulness of this approach to creativity training. The article is based on empirical data and our experiences from the Applied NeuroCreativity (ANC) program, taught at business schools in Denmark and Canada. In line with previous studies of successful creativity training programs the ANC participants are first introduced to cognitive concepts of creativity, before applying these concepts to a relevant real world creative problem. The novelty in the ANC program is that the conceptualization of creativity is built on neuroscience, and a crucial aspect of the course is giving the students a thorough understanding of the neuroscience of creativity. Previous studies have reported that the conceptualization of creativity used in such training is of major importance for the success of the training, and we believe that the neuroscience of creativity offers a novel conceptualization for creativity training. Here we present pre/post-training tests showing that ANC students gained more fluency in divergent thinking (a traditional measure of trait creativity) than those in highly similar courses without the neuroscience component, suggesting that principles from neuroscience can contribute effectively to creativity training and produce measurable results on creativity tests. The evidence presented indicates that the inclusion of neuroscience principles in a creativity course can in 8 weeks increase divergent thinking skills with an individual relative average of 28.5%.

## Introduction

We have discovered a new approach to train creativity: through the neuroscience of creativity. While the neuroscience of creativity cannot yet claim to be an operational research domain, we have in recent years been experimenting with applying the current advances and insights from neuroscience to increase the creativity of master level business students. In this article we will argue for the usefulness of neuroscience for creativity training, and support this claim with empirical data collected from the creativity training programme Applied NeuroCreativity (ANC).

Creativity is one of the most unique of human skills. It is thus important to develop more effective ways to train creativity, in order to create excellence and differentiation in any domain. Naturally, research on various approaches to enhance creativity is widespread, and well developed both in terms of creating the right conditions for creativity in education (see Selvi, 2007 for review) and for creativity training programs and their effectiveness (see Scott et al., 2004 for a quantitative review). In an impressive analysis of 70 creativity training studies, Scott et al. (2004) conclude that a fundamental understanding of the underlying concepts of creativity, combined with real life application, was the most effective approach to train creativity. Furthermore, they argue that the success of creativity training depends on a sound understanding of the critical components of creative thought.

We see the neuroscience of creativity as offering exactly that—a uniquely clear and sound understanding, through its tangible and rational conceptualizations of the cognitive processes involved in creative thinking. We have therefore constructed ANC, based on insights from existing successful creativity training programs, but with the inclusion of neuroscience as the underlying conceptualization. If the conceptualization used in creativity training is crucial for the success of the training, as concluded by Scott et al. (2004), and if neuroscience provides a clearer conceptualization, this approach should be a promising future direction for creativity training.

## Theoretical background

### Definition of creativity

It is impossible to write about, or teach, creativity without providing a sound theoretical definition of the concept of creativity. We will not dig ourselves and the reader down in the various understandings of creativity and their philosophical underpinnings, as this is continuously done more in-depth elsewhere (e.g., Glück et al., 2002; Klausen, 2010). Most researchers agree on what is considered the Standard Definition of Creativity (Runco and Jaeger, 2012): *Creativity requires both originality and usefulness*, as originally proposed by Stein (1953). In this paper, we rest our work on a more neurologically sound extension of the standard definition “… *the forming of associative elements into new combinations which either meet specified requirements or are in some way useful. The more mutually remote the elements of the new combination, the more creative the process or solution*” (Mednick, 1962, p. 221).

### Creativity training

The various studies related to enhancing creativity describe a range of ways to encourage or enhance creativity, and the two main approaches can be said to be through optimizing the creative environment (e.g., Moore, 1990; Westby and Dawson, 1995; Anderson and West, 1998; Ekvall and Ryhammer, 1999; Fatt, 2000) or creativity training (e.g., Feldhusen et al., 1970; Noller and Parnes, 1972; Nickerson, 1999). Educational researchers have been engaged in the topic of teaching creativity for decades (see Gregerson et al., 2013), but while this research is well developed in terms of creating the right conditions for creativity in education there is little focus on how to explain cognitive principles to enhance creative ability. Although the research regarding teaching creativity is important for facilitating creativity, we will in this paper focus on creativity training and the importance of the conceptualizations of creativity used.

For decades, researchers have been developing and testing numerous approaches to creativity training, and there exists a broad span in scope, teaching methods, purpose, length, and conceptualization of creativity. This has led to an extensive number of formats, and reviews of some of these can be found in Bull et al. (1995) and Smith (1998). In their qualitative review Scott et al. (2004) offer an in-depth overview of existing approaches, the various types of programs and their measurable successfulness. The main conclusion is that creativity training works, however course design has an important influence on the effectiveness thereof. The four critical aspects that seem to be particularly useful in successful creativity training are summarized as:

First, training should be based on a sound, valid, conception of the cognitive activities underlying creative efforts. Second, this training should be lengthy and relatively challenging with various discrete cognitive skills, and associated heuristics, being described, in turn, with respect to their effects on creative efforts. Third, articulation of these principles should be followed by illustrations of their application using material based on “real-world” cases or other contextual approaches (e.g., cooperative learning). Fourth, and finally, presentation of this material should be followed by a series of exercises, exercises appropriate to the domain at hand, intended to provide people with practice in applying relevant strategies and heuristics in a more complex, and more realistic context. (Scott et al., 2004, p. 383).

This approach is very similar to two of the most widely applied training programs: Purdue Creative Thinking program (Feldhusen et al., 1970) and the Creative Problem-Solving program (Noller and Parnes, 1972). Both these programs are based on a combination of first describing key cognitive aspects of creativity, before applying them in practice. In addition to the design of the training programs, the model of creative processes utilized is an important element in creativity training. Since Wallas’ (1928) well-known five-stage model (preparation, incubation, intimation, illumination and verification), a broad range of descriptive creative process models has been developed (e.g., Osborn, 1953; Sternberg, 1988; for review see Mumford et al., 1991). While the most common feature of creativity training is the widely acknowledged component of creative thought, divergent thinking (Fasko, 2001), the other components of creative thought and related processes emphasized in the existing courses vary.

Another, and perhaps more controversial, question for creativity training is the matter of domain specificity (for a summary, see Selvi, 2007). Can creativity training within one domain increase creative performance in other domains, or will the increase in creative skills only be related to the domain where it was taught? In a study of poetry creativity training Baer (1996) demonstrates that while the training worked for writing poems it did not improve the creativity in short story writing, and concludes that creativity training should be based on general domain independent concepts that are demonstrated in a domain relevant context.

While Bull et al. (1995) identified “cognitive approaches” as one of four general approaches to creativity training, Scott et al. (2004) distinguish between approaches based on “cognitive processes” and on “associational and affective mechanisms”. We argue that with the advancement in neuroscience these two can now be connected, by explaining the cognitive processes using the associational and affective mechanisms to make the cognitive concepts more accessible and tangible. To the best of our knowledge there exist no published studies of such an approach to creativity training. While many theories of creative thought are intangible and hardly related to practical implications, we see the neuroscience of creativity as a new framework that offers a uniquely clear perspective on creativity and creative tools—and at the same time a direct real world application for creative processes.

### Teaching the neuroscience of creativity

Creativity is a complex and multifaceted concept not easily defined nor understood. Understanding the neurobiological basis of creative brain processes requires not just an understanding of the concept of creativity, but also a thorough neurobiological background. However, although this represents a dilemma for teaching creativity with neuroscience, we have explored an approach we believe is capable of giving the layman student a simplistic, yet correct, understanding of the cognitive aspects of creativity through neuroscience. However, this has proven a delicate balance between giving too much and too little information. The way we have resolved this issue is by first giving a full theoretical background of the current state of what is known about creativity from neuroscience (8 h of lectures), starting from the single neuron level (e.g., lateral inhibition, the role of activating and inhibiting neurotransmitters, how these are regulated in different mental states and differences in creative individuals) to recent advances from neuroimaging studies (for recent reviews see Arden et al., 2010; Dietrich and Kanso, 2010). This is all explained in layman’s terms, and it is consistently pointed out what is important for comprehension and what is important for application. Even though this is still much too complex to fully grasp, it provides the student with a solid framework to ease the understanding and implementation of the brain processes introduced in the course.

All the theory is subsequently boiled down to a simplified and easily comprehensible model that we have labelled *NeuroCreativity*, consisting of five key concepts based on basic brain processes (priming, close and remote associations, inhibition, fixation and the release of inhibition—referred to as incubation) we see as being most important, in addition to well documented neurological processes needed to understand the neural processes of creative behavior. The main idea behind this approach is based on Mednick’s (1962) theory that creative processes can be understood as the ability to rearrange knowledge that already exists in the mind, and thus the greater the number of associations (especially remote associations) an individual has to the requisite elements of a given problem, the greater the probability of reaching a creative solution. Our theoretical framework is therefore a merge between associative theories of creativity and basic neuroscientific knowledge of how the brain’s associative networks function and their natural limitations (e.g., how the neurological principles of lateral inhibition can be used to understand cognitive fixation). A more detailed description and explanation of the course is beyond the scope of this article, and will therefore be presented in a later publication.

Greatly simplified, the practical implementation is grounded in understanding how the brain makes associations, and how differences at the level of information processing (both internally generated and externally perceived) can affect creative ability. For example, by understanding the basic mechanisms of how associations are formed, encoded, retrieved from memory etcetera, the direction, the amount and remoteness of associations can be manipulated. Other researchers have already observed that the amount or remoteness of associations can be manipulated during problem solving (e.g., Hofstadter, 1995; Clement, 2008). Our hypothesis is therefore that:

H1: A tangible understanding of the neurological underpinnings of creative thought, will produce measurable changes in trait creativity.

This hypothesis is well in line with previous studies demonstrating that explaining the nature of creativity is an effective part of creativity training (e.g., Speedie et al., 1971; Clapham, 1997) and forms the basis for this study. With the rapid development of the neuroscience of creativity, we see it as a natural next step to start using neurological conceptualizations in creativity training.

## The Applied NeuroCreativity course

The ANC is offered twice a year at Copenhagen Business School (CBS—Masters level, Denmark) and annually at Sauder Business School (UBC—MBA level, Canada). From 2013 it will furthermore be offered at the Technical University of Denmark (DTU—Masters level, Denmark). Each course runs for 8 weeks, with a weekly session of approximately 4 h of teaching and supervision. Furthermore, 2 to 6 h of independent project and theory work in groups between each session is expected. A total of 156 students have attended the course so far with an average of 39 per course (range 21–58). The entry requirement is 4 years of university studies, and the average age of the participants is 26 (range 22–44). The final hand-in is a written report reflecting on the creative process in relation to theory, and the participants are assessed based on an oral defence of this report.

The overall goal of the ANC course is simple: to improve trait creativity of the participants. The teaching philosophy is based on the metaphor of expertise as learning how to drive a car. To become an expert, one first has to understand and learn the mechanics of the car and the system of traffic. One does not have to be a mechanic, or a traffic analysis expert, but one has to understand the basic functions and the relationships between the two to effectively drive a car. When the basic principles are learnt, the learner will have to learn to apply the theoretical knowledge in practice, under close supervision. After a certain amount of practice, the learner is able to apply the knowledge without supervision, and from that point it is up to the participant to distil the skills into expertise.

The training focuses on four key aspects:

- Understand creativity through the neuroscience of creativity.
- Understand the difference between divergent and convergent thinking, and how the combination of these two is the source of creativity.
- Learn various creative tools, to understand why and how such tools work from a neurological perspective, and when to use them.
- Get practice with applying the creative tools in practice, while reflecting and (if necessary) act on the ongoing neurological processes, if these are limiting the creative process.

Throughout these four aspects ANC seeks to eliminate what we have experienced as the three main assumptions hindering creativity: (1) *I’m not a creative person*, (2) *I don’t know how to be creative* and (3) *I have no practice with being creative*. The neurobiology of creativity plays an all-important role for eliminating the first, through understanding that all humans have a physical potential for being creative, but it is also used to eliminate the two latter through the introduction, explanation and exploration of creative tools.

### Structure

The ANC course is designed based on the structure of existing successful creativity training programmes such as the Purdue Creative Thinking program (Feldhusen et al., 1970) and the Creative Problem-Solving program (Noller and Parnes, 1972). ANC makes use of what is described above as the key factors for the success of existing programmes: lengthy and challenging training, various domain related exercises, real world application of heuristics, and most importantly a sound conceptualization of cognitive principles underlying creative efforts. The content is structured in two parts: (1) a theoretical part giving the participants a fundamental understanding of the brain processes involved in creative thought (3 weeks), and (2) a practical part where this knowledge is sought applied through various creative tools used to solve a real world creative challenge for a major international company (5 weeks). The two parts are considered equally important in the course, and the structure aims at allowing the participants to unite theory and practice through collaboration and supervision, with the neurological knowledge being the red thread throughout the course. The weekly sessions after the theoretical part are based on “studio teaching” (Green and Bonollo, 2003), known from design and architecture schools, where short lectures are accompanied with exercises, tutorials and team based project work with supervision. In accordance with Baer’s (1996) findings on domain specificity, the training is domain specific but the underlying conceptualizations taught are domain independent.

After the theoretical part of the course the students design their own creative tool or strategy, which they believe will facilitate the newly acquired theoretical concepts in a creative process. This exercise is used to force the students to reflect on the real world applicability of the neurological concepts. They are then introduced to a range of classic creative tools (e.g., classical brainstorming, negative brainstorming/bad ideas, “what would Jesus do” and brainwriting), and the five key concepts of the NeuroCreativity model are used to explain why and when these creative tools can be helpful to overcome or aid a cognitive process, and what might be reasons for the tools not working in certain situations.

### Creative process model—the Double Diamond

**Figure 1:**
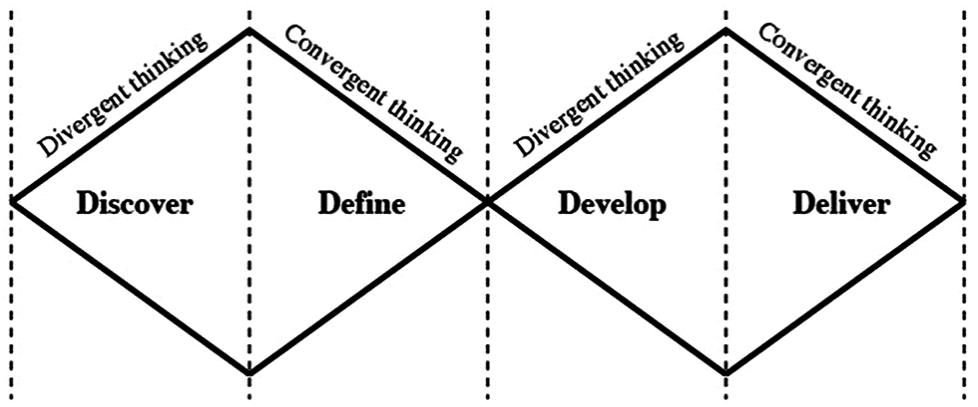
**The Double Diamond (DD)**.

Once creative thinking is understood, a key feature is knowing when to apply creative thinking and when to balance with critical thinking. While other creativity training programs focus on descriptive models of creativity, ANC utilizes the simplistic prescriptive Double Diamond (DD; Figure 1) model developed by the UK Design Council (Design Council, 2005), a model focusing solely on combining periods of divergent thinking with periods of convergent thinking, emphasizing that divergent thinking alone is not a sufficient condition for creativity training (Persaud, 2007).

Although divergent thinking and convergent thinking are separate cognitive processes these are of equal importance for creativity. We argue that divergent thinking can be thought of as a process that leads to novelty, and convergent thinking to usefulness. The participants are thus introduced to the DD, as one way of consciously seeking to combine divergent and convergent thinking to come up with a solution that is both novel and useful. As the ANC participants are master students with at least 4 years of university studies, we consider them well trained and experienced with conventional convergent thinking. Instead of rejecting this thinking process, the teaching emphasizes the importance of shifting between divergent and convergent thinking.

### Course evaluations

On finishing the course, all students were asked to rate the course through an independent assessment procedure carried out by the universities. Of the 156 participants, 71 filled in the evaluation (45.5%). The two universities CBS and UBC have slightly different course evaluation formats. The CBS form contains two questions considered related to the quality of the course, assessed on a 5-point scale from one “completely disagree” to five “completely agree”. ANC was rated to averages of 4.5 for “*The course has extensively increased my knowledge of the subject*” and 4.4 for “*My overall impression of the course is positive*”. The UBC form contains two questions considered related to the quality of the course, also assessed on a 5-point scale from one “strongly disagree” to five “strongly agree”. Here ANC achieved averages of 4.7 for “*The term projects (papers, assignment etc.) provided a useful learning experience*” and 4.8 for “*I would recommend this course to other students*”. For all four questions this gives an average of 4.6 out of 5.0. Unfortunately, neither of the universities disclose the average score of other courses offered, but these rankings are considered as unusually high and being in the top-end of the scale (Bo T. Christensen, study board chairman, personal communication). This indicates that the course design and execution in general is successful.

## Materials and Methods

In the following we will present two sets of empirical data investigating the underlying hypothesis and the effectiveness of this teaching method. The first data set is an isolated experiment with none-ANC students, investigating whether an ANC lecture on the neurobiology of creativity reduces the number of fixations in a classical design fixation task (the full study is published in Howard et al., 2013). The second set is a quantitative measure of changes in the participants’ divergent thinking skills before and after the ANC course, compared to a control group from two courses with highly similar design but without the neuroscience component. Lastly, we present the qualitative course evaluations from the participants, as an indicator for what might cause the effects shown in the analyses. This focuses on self-reported value of the neurological knowledge and perceived increase in creative ability.

### Fixation

As part of the process with testing the underlying hypothesis, we have started a series of experiments outside the ANC course context, where we investigate the impact of each of the five key concepts independently. In the first of these experiments (Howard et al., 2013), we isolated the concept of fixation in a controlled study with engineering students, to investigate whether knowledge of the neurological concept of fixation would decrease the number of fixation in a classical *design fixation* task. Twelve teams of four engineering students were challenged with three different design tasks, directly adopted from Jansson and Smith (1991) seminal study on design fixation. Each of these design tasks has built-in elements that initiates fixation, and is constructed so the amount of design fixations in the final solutions can be calculated. The subjects were explicitly told to avoid these design flaws. The experimental procedure was structured so that all groups conducted a design task before and after an ANC lecture of the neurobiology of creativity, including fixation as one of many concepts, but without knowing that they were solving a design task facilitating fixation, i.e., the true purpose of the experiment. The order of the three design tasks was counterbalanced between teams to account for differences in task difficulty. The lecture was framed as a lecture in the neuroscience of creativity from the ANC course, and fixation was only explained as a consequence of how the brain makes associations. One of the hypotheses behind this experiment was that an understanding of the associative brain processes causing fixation would decrease the amount of fixations in a real design task. Without any prior knowledge of fixation the students generated a total of 86 designs (7.2 per team) with a total of 76 fixation elements (fixation ratio: 0.88). With insights into associative processes, 89 designs were generated (7.4 per group), but with a significant drop in fixation elements to 48 (fixation ratio: 0.53; *p* = 0.026). For a complete analysis see Howard et al. (2013).

### Assessing measurable changes in trait creativity

The second set of empirical data investigates whether there are measurable changes in trait creativity, measured as divergent thinking in participants, following the 8-week intensive ANC course. This is compared to a control group of students participating in two highly similar elective courses, but without the introduction to neuroscience. Divergent thinking was measured before the first lecture, and again 8 weeks later with the Alternative Uses Test (Guilford, 1967). In this test, participants are instructed to write down as many alternative or unusual uses for a common object. Besides being the most widely used test of creativity (Davis, 1997; Cropley, 2000), used in approximately 40% of all studies with college students and adults (Torrance and Presbury, 1984), several studies have documented its test-retest reliability (e.g., Mackler, 1962; Yamamoto, 1963) and it is a recommended effectiveness-test of efforts when teaching students how to think more creatively (Dehaan, 2011).

#### Participants

One hundred and forty seven students from CBS participated in the study: 83 students from the ANC courses (fall 2012 and spring 2013) and 64 from the two non-neuro creativity (NNC) courses (spring 2013). However not all participants were present at both tests. Only those that completed both tests were included in the analysis. In total, 99 students performed both tests (62 from the ANC-courses and 37 from the control NNC-courses).

#### The NNC control group

As a perfect control group hardly exists in this line of research, we have sought to find a control group with as many similarities as possible but without the neurological conceptualization. As the ANC course, the NNC control courses consisted of two parts—one theoretical and one practical part. While the ANC theoretical part is focusing on a neurological conceptualization of creativity, the NNC courses depart in classical cognitive theories of creativity. Where ANC goes more in depth with a neurological understanding of the cognitive principles of creative thought, the NNC courses are linking the cognitive aspects to artistic and organizational understandings of creativity. Apart from this fundamental difference, the two course types are similar both in structure and in content. All courses were elective master level courses at CBS, taught in the same studio, using studio teaching and both with a duration of 8 weeks in the same period. Furthermore the NNC courses introduce the DD as the process model, with equal amounts and duration of divergent and convergent phases, and utilize an external real life creative challenge from a major international company.

#### Materials and procedure

Based on the Alternative Uses Test, participants were instructed to write down as many unusual uses they could possible think of for a given common object, and were told not to include ordinary or unrealistic uses for the object. Prior to all tests an example was given for allowed and disallowed uses for an example object, *Paperclip*. Ordinary use: hold paper together; unusual use: use as an earring; unrealistic use: fly it to the moon. In each test subjects were given three common objects and 3 min for each object. Across all tests the following six objects were used: *Brick*, *Newspaper*, *Pen*, *Car tyre*, *Towel* and *Shoe*. The ordering of the common objects was counterbalanced between courses and tests, so that the two ANC courses had the opposite three objects in the first and second test and the same with the two NNC courses. As many students at CBS are international students, subjects were instructed to write in their native language, and after testing all subjects scored their own test by counting the number of ideas for each of the objects.

## Results

**Figure 2:**
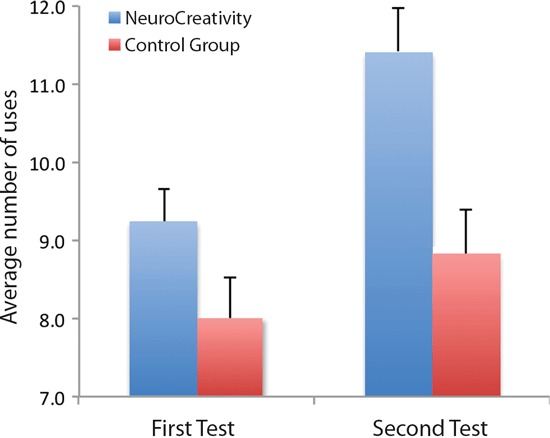
**The average number of uses generated per object on the first and second test for each group. Error bars represent the standard error of the mean**.

We used the traditional measure of creativity, which is the number of uses generated in the Alternative Uses Test (fluency) (Glover and Gary, 1976; Eisenberger et al., 1998). Although other performance measures on this test can be measured (originality, flexibility and elaboration), fluency accounts for almost all of the variance on divergent thinking tests (Plucker and Renzulli, 1999). For both the ANC and control courses the distribution of number of ideas generated was normally distributed in both tests (ANC test one: mean = 9.2, SD = 3.3; test two: mean = 11.4, SD = 4.2; NNC test one: mean = 8.01, SD = 3.16; test two: mean = 8.8, SD = 3.3). Using a mixed model repeated measures ANOVA with Course (ANC vs. control) as between participants factor and Time (pre vs. post training) as within participant factor, we found a significant main effect of Course between subjects [*F*(1, 97) = 7.58, *p* = 0.007] and a significant main effect of Time within subjects [*F*(1, 97) = 20.2, *p* < 0.000] as well as a significant interaction between Course × Time [*F*(1, 97) = 4.2, *p* = 0.043]. However, the two groups did not have the same initial starting point, indicating that the sampling of subjects between the two groups were not truly random. This was tested using a simple paired *t*-test of the pre-training scores of the two groups (*p* < 0.000). Due to this, we performed an analysis of covariance (ANCOVA) with Gain scores as the response, Course as design factor and the pre-training test as a covariate, thereby taking initial differences in pre-training test scores into account. Even after adjusting for differences in pre-training scores we still found a significant difference in gain scores between the two groups [*F*(2, 96) = 4.01, *p* = 0.0212, η^2^ = 0.5], with ANC showing the highest gain (ANC gain score = 2.2, SD = 3.5, NNC gain score = 0.8, SD = 2.5), meaning that students in the ANC course had a significantly higher increase in fluency after the 8 week training period compared to the control NNC courses. On the individual level, this constitutes an average relative increase in trait creativity of 28.5% for the ANC students. For the NNC control group there was a slight increase between the first and second test but not enough to reach significance (two-tailed paired *t*-test; *p* = 0.06). While this increase may have reached significance with a larger sample size, it was still significantly lower than the ANC participants as shown by the ANCOVA. The results from the two fluency tests are shown in Figure 2 below.

**Figure 3:**
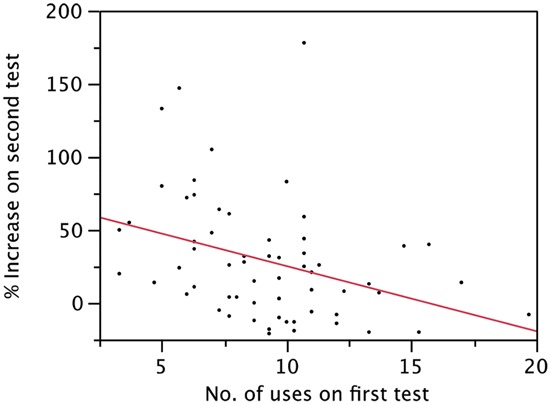
**The negative correlation between individual increase calculated in percentage on the second test and performance on the first test**.

Using linear regression we found a relationship between initial level of trait creativity, and the individual increase (measured in percentage) in divergent thinking on the second test, showing that the lower the initial level of divergent thinking, the higher the relative increase (*r* = −0.35; *p* = 0.0047; Figure 3). This suggests that participants starting off with a low level of trait creativity had most to gain from the course. However, it should be noted that there is a natural ceiling effect in this test due to the time constraint of the test (3 min per object). This evidently means that if writing speed is kept constant, after a certain number of ideas are reached the time constraint might be the limiting factor rather than trait creativity.

### Qualitative course evaluation

While it is repetitively shown that various forms of creativity training work, the question of why it works remains unresolved. For the dedicated training programmes alone, aspects such as providing new strategies (Mumford et al., 2003), increasing motivation (Birdi et al., 2012) and training thinking skills (Birdi, 2005) are considered. For the teaching environment Selvi (2007) uses 18 existing studies to list as much as 28 factors said to influence creativity in educational environments. To get a better understanding of what might be reasons for the positive effect of ANC, we have consulted the anonymous course evaluation collected by the universities. It should be stressed that due to the limited number of responses this qualitative data is merely considered an interesting pointer to whether the neuroscience component played an important role, and whether the students perceived an effect on their creativity. Of the 71 participants filling in the evaluation, 63 wrote additional comments for the course (88.7%), and we have organized these comments in three categories: *Neuroscience* (any comment regarding the neuroscience content of the course), *Effects on creativity* (any comment not in the first category, related to perceived effects on creativity and real life applicability) and *Other* (e.g., curriculum, work load, exam format). In the *Neuroscience* category there were 13 quotes (20.6% of all comments) and all were positive. An example of such a comment:

I love the way the course is built up! First of all getting a pure neurobiological insight into creativity and afterwards getting to practice the knowledge you’ve just learned, and deepening ones awareness of mechanisms that take place during creative processes and slowly learning how to handle it.

The *Effects on creativity* category counted 10 comments (15.9%), and again all were positive. An example of a comment in this category:

A unique course which increased my level of creativity and made me aware of relevant aspects that happen on a daily basis. This course also enabled me to use what I learned outside the classroom, by applying this knowledge at work or at home.

The category *Other* contained the 40 remaining comments (63.5%) which were not considered relevant for evaluating the use of neuroscience, creativity or training of creativity. The participants were not asked about the usefulness of neuroscience, or effects on creativity, thus the fact that they freely chose to positively comment on these two topics is considered an affirmative indicator for both the usefulness of neuroscience and the impact on participants’ creativity.

## Limitations and future research

When studying educational training, there are always more variables than one can control precisely, due to the complexity in learning processes and constraints within educational systems. One approach is to move back into the laboratory, but then we usually lose the ability to study larger numbers of subjects and some ecological validity, or the ability to trust findings as applying to real world educational situations. So as in the business world, one must often settle for the best control groups available, rather than perfect controls. One way to make up for these limitations is to triangulate from multiple sources of data that speak to the same overriding issue from different angles, as we have attempted here. With that being said, there are still important limitations to our studies. In the fixation study (Howard et al., 2013), there was no control group and the results should thus be treated with caution, as we cannot rule out practice effects. The decrease in fixation could be due to the effect of solving a second design problem more than the ANC lecture. However, this seems improbable as fixation effects are known to build up over time (the more one works on a problem, the higher the level of fixation) (Diehl and Stroebe, 1991; Nijstad, 2000) and we would hence expect an increase in fixations in relation to practice effects. Also, as all of the participants were engineer students and experienced with solving these kinds of design problems, we would expect practice effects in this regard to be minimal. Fixation occurs more often with examples that are typical and familiar in respect to a designer’s background (Perttula and Liikkanen, 2006). Nonetheless, it still remains possible that the decrease in fixation found after an ANC lecture on the neurological theory of how we make associations, could be due to mentioning the phenomenon of fixation and thereby steering the student’s attention to it. Even so, the subjects were explicitly told to avoid the design flaws in the example designs, both before and after the lecture, and thus their attention to the fixation features in the design examples should be comparable. Although their attention may have been steered towards the concept of fixation, this effect alone does not seem to be able to account for the decrease in fixation. Previous research has shown an inability to avoid fixation effects at the conceptual level, also known as cryptomnesia or unconsious plagiarism (Brown and Murphy, 1989), for engineer students (Perttula and Liikkanen, 2006) as well as for professional designers, even those that teach design on a regular basis and thus are familiar with the concept of fixation (Linsey et al., 2010).

In the second data set the NNC control group showed a lower initial starting point in divergent thinking skills. We have no indication of any discernable factors that biased some students to take one course over the other, or discernable differences in educational or personal backgrounds. Still, with the current data set, we cannot determine whether the pre-training difference was simply due to chance, the lower sample size in the control group, or even factors such as motivation. Therefore, our results could be affected by other factors than the neurological conceptualization, although this appears to be the only essential difference between the groups. Future studies should include how well each participant understood the neurological content of the course, and include groups with and without practical training, as this could be the next step towards determining the true contribution of the neurological conceptualization of creativity.

## Discussion and conclusion

In this paper we have investigated the validity and effectiveness of using neuroscience as a framework for creativity training. While previous research has shown that successful training of creativity can be achieved through first teaching the underlying concepts of creativity and then how to apply these in a real world context, no study has, to the best of our knowledge, previously used neuroscience as a framework for training creativity. Our findings support the hypothesis that a tangible understanding of the neurological underpinnings of creative thought improves the divergent thinking aspect of creativity.

The ANC course is based on a simple principle: the more one understands about the basic ways our brain functions in relation to creativity, the more one is able to utilize ones full creative potential. Testing this, we found a significant increase in divergent thinking measured at the first day of the ANC course and again at the last (period of 8 weeks), using the Alternative Uses Test. This test was also performed on a control group from two highly similar courses, where the essential difference is considered to be the use of neuroscience as conceptualization. At the individual level participants in the ANC course had an average relative increase in divergent thinking ability of 28.5% after completing the course, while we found only a small and non-significant increase in the scores of the control group. Analyzing the covariance we found that the increase in fluency in the ANC course was significantly higher than that of the control group, even when adjusted for differences in pre-training scores. These results demonstrate that a thorough conceptualization and understanding of the neurological activity underlying creative thought can indeed influence cognitive performance. This was shown for the divergent thinking test, as well as for the realistic design tasks in the fixation experiment (Howard et al., 2013). This study investigated design fixation in engineering students, and similarly demonstrated that specific knowledge of the neurological basis for how we make associations (20 min lecture) could in an experimental setting significantly reduce the number of fixations in realistic design tasks (ibid).

Previous studies of creativity training programs have been criticized for lacking internal and external validity (Cropley, 1997; Nickerson, 1999). The tests used are often very similar, or identical, to the training material. To ensure internal validity we used a divergent thinking test where participants generated unusual uses of common objects, a task that is unrelated to the content of the training. The ANC course focuses on combining divergent and convergent thinking, and do not provide any training designed specifically for improving performance on divergent thinking tests. Furthermore, no creativity tools or methods taught in the course are designed to generate unusual uses for common objects. The external validity issues in similar studies are related to sample type and size, as studies are often from school settings with small groups of young students, blurring the transfer value to other settings. We used a large sample of final year master students, and domain specific training with a real world challenge, while the effect of the training was assessed with a domain independent test.

As divergent thinking tests are often wrongly referred to as a measure of creativity, we must emphasize that these kinds of tests are only a measure of the potential to be creative. Although this is an important indicator of creativity, it must not be confused with actual creativity. Divergent thinking is one of many components of creativity, as there is much more to creativity than the number of ideas one can produce. Creative ability is generally believed to be equally based on knowledge and analytic thinking. For example Sternberg and Lubart (1992) argued that creativity is a function of six factors: intelligence, knowledge, thinking style, personality, motivation and environmental context. However, divergent thinking does have its validity in assessing important aspects of creative ability and in this case changes to that ability. According to Guilford (1959) and many others, divergent thinking provides the foundation for creative production, as it requires ideational searching without directional boundaries. Similarly, Robinson (2001) has argued that divergent thinking is the “essential capacity for creativity”. This is in line with the philosophy of the course, as the point is not to “become creative”, but instead learning to fully utilize ones existing divergent (and convergent) thinking skills—and solely based on how the comprehension of the neurological underpinnings increase ones creative potential. The underlying assumption of this hypothesis is that creativity is a natural component of human thought, one that we all have to a varying degree, but one that not all have learned to harvest. This view has been confirmed by an impressive study where 1600 children were given a divergent thinking test at age five and again at age ten and 15. Compared to 280,000 adults, 98% of the children started out at creative genius level, which rapidly decreases with age towards the adult level (2%) (Land and Jarman, 1992). As these authors pointed out it seems that “non-creative behavior is learned”. Our results support this postulation, as one would predict that if creativity were the natural baseline, those who had “un-learned” creativity the most, would also show the greatest increase—returning towards the natural baseline. Indeed, we found a negative correlation between initial level of divergent thinking ability and the individual relative increase on the second test, indicating that those with the lowest levels of trait creativity had most to gain from the course. In fact, we believe that the neurological knowledge in the ANC course plays an important role in convincing the participants that they each hold the physical potential to be creative, and thus a change in perception and related self-esteem could potentially account for some of the improvement. The following quote from the qualitative feedback outlines this nicely:

The knowledge of neurobiology (i.e., neuroscience), in my view is very useful. Firstly, it cleans my long-held misunderstanding regarding creativity and genetics. Secondly, the facts and information learned during the classes are important in my future discussion on creativity with others. I believe it is important to educate people to understand that creativity is not all about genetics and innate intelligence. Lastly, the study leads to personal encouragement to find ways to improve my creativity.

It is also considered a promising indicator that 20.6% of the qualitative comments were uninvitedly reporting positively on the neuroscience aspects. And considering neuroscience as the only essential difference between ANC and NNC, the significant difference in increase of trait creativity between the two imply that neuroscience is a crucial element. We see the evidence presented in this paper as indicating that the neurological conceptualization used in the course plays a crucial role for the positive effect of the training.

Still, as with other successful creativity training programs, the question of exactly why creativity training works and in this case the neural aspect of ANC, cannot be answered with the current data set. However, based on our experience and conversations with students, there seems to be several contributing factors. As mentioned, one that is often brought to our attention and seemingly of most importance is the insight that the creative process from a neural perspective is innate (in the prewired sense) and as natural as breathing. Several students have even reported this as a life changing realization, as many start the course with the assumption “*I’m not a creative person*”. On the other hand, some students may hold the belief that they are creative, but then do not appear to know how to be creative or are confused on how to structure the creative process. Thus, although the enhancement of creative self-efficacy might be the main aspect in the success of ANC, it is more likely an interplay of learning to trust ones own creativity and knowing how to utilize that creativity and attaining creative experience. This combination could conceivably ease creative task performance, hence the fluency of ideas. However, although these achievements are of apparent importance for creativity, they do not seem unique for the ANC course. The control NNC course presumably provide similar comprehensions, if not through neuroscience at least through experience from working creatively in the course. Nonetheless, it may be that a change in meta-knowledge of creative processes can alter the way people approach creativity tasks, and that the neural conceptualization used in ANC is more efficient at providing this. Ironically, the explanation of why the neural approach in ANC is more successful is then perhaps found at a more neurobiological level.

Scott et al. (2004) emphasize the importance of sound conceptualizations of creativity for successful training. In our point of view there is no more sound and rational conceptualization for creativity than the neurological mechanisms underlying creative thought.

## Conflict of interest statement

The authors declare that the research was conducted in the absence of any commercial or financial relationships that could be construed as a potential conflict of interest.
